# Swedish Efforts to Contain Antibiotic Resistance in the Environment—A Qualitative Study among Selected Stakeholders

**DOI:** 10.3390/antibiotics11050646

**Published:** 2022-05-12

**Authors:** Ingeborg Björkman, Marta Röing, Jaran Eriksen, Cecilia Stålsby Lundborg

**Affiliations:** 1 Department of Public Health and Caring Sciences, Health Services Research, Uppsala University, 751 22 Uppsala, Sweden; marta.roing@pubcare.uu.se; 2 Department of Global Public Health, Karolinska Institutet, 171 77 Stockholm, Sweden; jaran.eriksen@ki.se; 3 Department of Infectious Diseases/Venhalsan, Stockholm South General Hospital, 118 83 Stockholm, Sweden; 4 Health Systems and Policy (HSP): Medicines, Focusing Antibiotics, Department of Global Public Health, Karolinska Institutet, Tomtebodavagen 18A, 171 77 Stockholm, Sweden; cecilia.stalsby.lundborg@ki.se

**Keywords:** strategic action plan on antibiotic resistance, Swedish stakeholder perceptions, One Health, qualitative study

## Abstract

Antibiotic resistance is a serious global threat to human and animal health. In this study, we explored perceptions of work to contain antibiotic resistance with a focus on the environment. Nine stakeholders from six different areas were interviewed in 2018. A short information update was given by informants from four of the areas in 2021. Interview transcripts were analyzed by conventional content analysis. The stakeholders’ perceptions were concluded in three categories: “examples of actions taken to combat antibiotic resistance”, “factors influencing work”, and “factors hindering work”. All informants reported having a role to play. Some of them were very engaged in this issue, whereas among others, antibiotics and resistance were just one part of a general engagement. To be able to act, the policymaker stakeholders asked for more knowledge about antibiotics in the environment and possible actions to take. Actions from the government were requested by several informants. Coordination of the work to combat antibiotic resistance in the environment was not recognized and the One Health approach was known at policy level but not among practitioners. Still, actions seemed to be coordinated, but this was, according to the stakeholders, based on findings from research in their area rather than on strategies developed by national authorities.

## 1. Introduction

Antibiotic resistance is a global threat to human and animal health [1,2]. The number of deaths at the global level associated with bacterial antimicrobial resistance in 2019 was estimated to 4.95 million [3]. The World Bank has described antibiotic resistance as a major threat to the world economy [4]. Antibiotic resistance can affect multiple sectors in society, as resistant bacteria can be transmitted between humans, animals, and the environment. Coordinated action has been suggested as a means of combating the threat of antibiotic resistance [5]. In 2015, the World Health Organization (WHO) published a Global Action Plan (GAP) based on a “One Health” approach [6]. The GAP set out five strategic objectives and stressed coordination between sectors and actors [6]. Member states were expected to develop and implement national action plans aligned with the GAP objectives [7]. The commitment of actors and stakeholders, for instance government authorities, policy makers, healthcare workers, university teachers, pharmaceutical companies and consumers, is essential [5], as well as multisectoral involvement, including human and animal health, together with the environment, trade, intellectual property, and innovation [8].

The Swedish government published its first action plan in 2016 [9]. When presented, it was based on a One Health approach, and had the overarching goal “To preserve the possibility of effective treatment of bacterial infections in people and animals”. A quotation from the foreword reads, “It is a top priority for Sweden that the action plan is put into practice”. This action plan was updated in 2020 [10]. Some years before the first action plan, in 2012, a national intersectoral coordinating platform was established to coordinate the work of national government agencies [9]. At that time, 21 government agencies were included in the platform [9], and some years later the number of government agencies involved was 25 [10].

This study is part of the ABRCARRO (A One Health Systems and Policy Approach to Antibiotic Resistance Containment: Coordination, Accountability, Resourcing, Regulation and Ownership)—an international project which aims to explore and describe how national action plans against antibiotic resistance were developed, implemented, monitored, and evaluated in Sweden, South Africa, and Swaziland. The project includes interviews with different categories of stakeholders, policymakers at government level, and professionals in human, animal, and environment/agriculture sectors, as well as policy document analyses. In this paper, we explore efforts to contain antibiotic resistance in the environment in Sweden.

Studying how actors and organizations work to contain the spread and development of antibiotic resistance in the environment is challenging. To begin with, it is known that the use of antibiotics has accelerated the development and spread of resistance in microbial populations [11]. However, the understanding of the role of the environment in the development and spread of antibiotic resistance is still limited [12]. At first, the role of the environment was recognized as a pathway for the spread of antibiotic resistance [5]. Later studies suggested that the environment is also involved in the development of antibiotic resistance [11,12]. Residual concentrations of antibiotics and resistant bacteria from human waste, animal waste, and manufacturing waste may end up in the environment [13]. Studies suggest that lakes can harbor antibiotic resistance genes, and also genes responsible for mobilization of genetic material [14].

Secondly, the problem of working to contain antibiotic resistance in the environment is multifaceted in such a way that multiple sectors must be involved to have some sort of impact. To reach effectiveness, organizations and actors generally not linked to each other most probably need to work together for the same goal. Research on how this can be experienced is sparse. Thus far, knowledge of actual Swedish efforts to contain antibiotic resistance in the environment, and how they are perceived, is limited. Further exploration is needed.

The aim of the present study was to explore and describe how informants working on environmental issues in a strategic selection of health and public sectors in Sweden perceive their own work, as well as other work in Sweden, to contain antibiotic resistance in the environment.

## 2. Method

### 2.1. Design

According to the explorative approach of the topic, a qualitative research design based on interviews was chosen [15,16]. By asking open-ended questions and letting the informant speak freely about the topic, data were collected as text, i.e., transcripts of the interviews. A content analysis approach was then chosen in the analysis and presentation of the findings of the transcribed interview texts. We used conventional content analysis with no predefined categories [17].

### 2.2. Informants

Our aim was to include informants involved in work to contain antibiotic resistance in the environment. We therefore approached individuals working at six strategically selected health and public service areas, see Table 1. Exploring their involvement and contributions can give insight into this work from their perspective, impart knowledge of the current situation in their area, and indicate directions for future efforts. Representatives from these selected areas were contacted by email and asked to participate. All approached persons accepted. In one case, the pharmaceutical company employee, the person first had to ask management for permission to take part in the interview. However, one informant withdrew the interview some years later. This was when the informant was asked to provide an update of the information (see below), explaining that they had no time to present an update and therefore chose not to participate at all.

### 2.3. Data Collection

A semi-structured interview guide was used, based on an interview guide previously used by the research group when studying perceptions of antibiotic resistance work in the human healthcare sector. Some questions were adapted to the purpose of the present study. The interview guide was first pilot tested on two informants working in the human and animal sectors, respectively. Results from these studies are presented elsewhere [18,19]. The main questions are presented in Table 2. The complete interview guide is available as Supplementary Material File S1.

During the interviews, informants spoke freely and shared their thoughts and understandings. The interviewer followed up with different probing questions, depending on what the informant was telling, for either more information or clarification. Author IB conducted the interviews during the period April to June 2018 at a place selected by the informant, usually at their workplace. The interviews lasted on average 47 min, with a range of 25 to 72 min. All interviews were tape recorded and transcribed verbatim by another person, and then the transcripts were checked and corrected, if necessary, by IB before the analysis process started.

### 2.4. Data Analysis

An inductive approach was used, with no predefined codes or categories. Author IB conducted the analysis, supported by author MR who acted as co-reader. The interviews were first read through to get an overview of the content. Next, interviews were read line by line and meaning units were picked out, given codes, and condensed. At this point each meaning unit and code was given an id-number to facilitate the analysis process. Meaning units were then sorted based on the codes, codes were merged and renamed in repeated steps, and codes that did not concern antibiotics or antibiotic resistance were taken away. Thereafter, all transcripts were sorted based on the new codes, and the content of each code was examined. 

The codes were rearranged in subcategories and three main categories were chosen in a final step to organize and present the content of the interviews as follows: Informants’ perceptions of actual efforts to contain antibiotic resistance in the environment, their perceptions of factors influencing their work, and factors hindering their work. All findings describe informants’ perceptions. 

### 2.5. Ethical Considerations

Ethical approval was sought from and granted by the Regional Ethics Board in Stockholm (Reg number: 2017/1999-31).

### 2.6. Contact with Informants in Autumn 2021 for Information Updating

Data collection took place in 2018 whereafter the interview material was analysed. However, the manuscript was not completed for a period of nearly four years. Thus, to be able to present current experiences, the authors felt that an update of the material would be of value. All informants were therefore contacted by email in the autumn of 2021. The aim was to obtain knowledge about the informants’ experiences and engagement in efforts to contain antibiotic resistance, and if they differed or were similar as to the time of the first interview in 2018. To help recall what they had said at the first interview, each informant was shown the findings generated from their interview. The informants were asked three questions: (1) whether the informant or their organisation worked in the same way as they did in 2018, or if it had changed, and if so how; (2) whether the informant had noted any new function or organisation which coordinated work against antibiotic resistance development and spread in the environment; and (3) whether there were any changes in cooperation in the work to contain antibiotic resistance. The answers to the email questions are summarized at the end of the results section.

## 3. Findings

We present our findings in three main categories (Table 3). They are further described and illustrated by quotes from the interviews as follows. 

### 3.1. Informants’ Examples of Actions Taken to Combat Antibiotic Resistance

The informants talked about many different actions, which they thought could contribute to combat antibiotic resistance. These actions were identified as subcategories in our analysis. Due to the large differences in work areas involved, and for a better understanding of the contribution of each specific area, the following presentation of the content of this subcategory is sorted according to the informants’ areas of work.

#### 3.1.1. Actions Taken by Government Authority

##### Subcategories: Monitoring Environment Samples

The informant from the Environmental Protection Agency declared the agency had only recently become involved in this work and had so far found it difficult to find its role. The task of the agency was, according to the informant, to describe the state of the environment by sampling water, sediment, fish and wild animals, but antibiotics and antibiotic resistance were not a major issue.

“*This cooperation between agencies started a few years ago, it was around then that some people started to talk about it […] and since then, it has grown […] although just here it is really not a major question*.”

(M2)

The informant gave examples of networks and platforms the agency was engaged in, both nationally, at EU level, and globally. According to this informant, antibiotics or antibiotic resistance were seldom the main focus in the environmental networks.

#### 3.1.2. Actions Taken in Medical Microbiological Research

##### Subcategories: Developing Knowledge, Spreading Knowledge, Action for Restrictive Antibiotic Use

The medical research informant expressed explicit engagement in antibiotic resistance issues, and creating new knowledge in this field, for example, studying how genes are exchanged, and how resistance is spread through plasmids and viruses. We know that genes are spread by plasmids, the informant explained, and we do know that resistance can be transferred, but we do not know to what extent. The researcher informant furthermore described a role as a teacher at a medical school, teaching medical students to always think carefully before choosing infection treatment, and to always refrain from antibiotics when possible. To contain antibiotic resistance in the environment, as well in humans and animals, both safe use of antibiotics, and decreasing the use of antibiotics was required, stated the informant.

“*I understand if you have, for example, a blood-poisoning and someone who is acutely ill, then I fully understand, it is clear then that antibiotics should be used I think, because then it is a matter of saving lives, it is the grey areas where I think you have to be more careful*.”

(M9)

#### 3.1.3. Actions Taken by Pharmaceutical Companies

##### Subcategory: Action for Restrictive Antibiotic Use

Safe antibiotic use, with narrow-spectrum antibiotics, and decreasing the use of antibiotics was brought up by the pharmaceutical company informant as ways to contain antibiotic resistance. It was therefore important to focus on keeping narrow-spectrum antibiotics commonly used in Sweden, as well as a wide assortment of dosage strengths and package sizes. According to this informant, individual adaption of antibiotics dispensed to patients, making sure the quantity of prescribed antibiotics does not exceed treatment duration, was fundamental for safe use and optimal antibiotic treatment.

“*Our most important task is, right now, to maintain a large assortment and a wide range. Which is the basis for being able to eh, treat optimally and not drive resistance*.”

(M10)

This informant noted that the company had been involved in starting up a multisectoral collaboration platform which gathered representatives from both healthcare and authorities, aiming to ensure access to antibiotics in Sweden. The company’s interest here, the informant continued, was to enable discussions about which antibiotics the companies should fight to keep and develop, and which strengths and package sizes to focus on.

#### 3.1.4. Actions Taken by Pharmacies

##### Subcategories: Action for Restrictive Antibiotic Use, Reduce Antibiotics and Bacteria Reaching the Environment, Spreading Knowledge

The pharmacy informants stated that decreasing the use of antibiotics, as well as safe use of antibiotics, were necessary to contain antibiotic resistance. Staff at pharmacies have a role here, informants said, and this was to provide patients packages of antibiotics adapted to their prescription, i.e., not give extra tablets that may be kept and used later for self-treatment, and by counselling patients on medication use and relief of adverse effects.

“*You should be given an adapted amount to take home” […] “yes, we also sell lactic acid bacteria, and some can help during an antibiotic treatment, counteract diarrhoea and such that could be an obstacle to completing the cure, so it can actually help someone complete their treatment*.”

(M6)

Furthermore, the pharmacy informants brought up the fact that pharmacies have systems to collect medical waste, where all kinds of medicines are collected and sent for destruction, with no special focus on antibiotics. One informant worked in a pharmacy chain which had employed a specific person to work with environmental aspects of medicines, including antibiotic production. This chain was deeply involved in the issue of antibiotic waste at antibiotic production sites, and tried to reach the public, politicians and authorities through seminars, podcasts, YouTube films and newspaper articles, hoping to create debate. Another activity mentioned by the informant was a campaign driven in cooperation by pharmacy chains, directed to the public, to not take antibiotics for common colds.

#### 3.1.5. Actions Taken in Hospital Environment Department

##### Subcategories: Reduce Antibiotics and Bacteria Reaching the Environment, Action for Restrictive Antibiotic Use

Hospitals have systems to manage medical waste, and the hospital wastewater is connected to the city wastewater treatment system, said informants working in this area. Medical waste, which included antibiotic waste, was collected in separate bins by the ward staff, and then sent for incineration. According to one hospital informant, the hospital environmental department focused on writing routines for collection, sorting and management of medical waste, and following-up whether they were adhered to by staff. 

The hospital environment department was responsible for creating lists of approved products to be used in the hospital, one informant reported. One example of a product that the informant especially did not want in use at the hospital was detergents with silver, since, according to the informant, bacteria can develop resistance to silver, which can lead to increased antibiotic resistance. This hospital environmental department furthermore cooperated with the hospital’s local Strama-group (Strama is the Swedish strategic programme against antibiotic resistance) in their efforts to reduce the use of fluoroquinolones. Another task of the environmental department, according to the informant, was to set environmental demands when ordering hospital food. The informant believed buying ecological meat was a way to support lower antibiotic usage in food-animal production.

“*There are certain substances, antibiotics that are extremely persistent in the environment, including fluoroquinolones that have long half-lives […] because we see a connection to the external environment or that effect, we have seen that, yes it makes sense to raise it as an environmental goal as well, so we can pursue this together*.”

(M8)

#### 3.1.6. Actions Taken in Wastewater Treatment

##### Subcategories: Reduce Antibiotics and Bacteria Reaching the Environment, Developing Knowledge

Wastewater treatment plants did not have a special focus on antibiotic resistance, according to informants working at these plants. Both informants believed that the use of high-quality wastewater treatment methods can contribute to containing antibiotic resistance. Both said they worked at plants which had decided to study and install new technology to improve water treatment, methods that reduce the discharge of bacteria. One plant was installing effective membranes, and the other ozonation technology. The latter method will also reduce drug residues, according to the informant.

“*We had a project just a couple of years ago then, or yes, it ended well last year, […] but then we had, among other things, sampling up there, where we looked at bacteria, how much bacteria comes out of the membranes and how much antibiotic-resistant bacteria comes out. And it was basically zero*.”

(M1)

“*Ozone is a very powerful eh, oxidizing agent, it breaks down most drug residues eh, or drug molecules, eh, quite effectively. […] It has a very strong effect on bacteria as well, they break down, so it is very effective*.”

(M5)

Both informants described how sludge was managed in the plants, since sludge, after the treatment process, and control of salmonella, metal, and toxic products, was often used in agriculture. Most antibiotics coming to the wastewater treatment plant stick to the sludge, said informants, and were thus separated from the water. Monitoring was also conducted at city wastewater treatment plants, informants said. In one treatment plant, follow-ups focused on known toxic compounds and on bacteria. The other treatment plant had decided to study pharmaceutical residues with the highest concentration levels in wastewater, the informant reported. However, this did not include antibiotics.

### 3.2. Informants’ Perceptions of Factors Influencing Work

#### 3.2.1. Organisational and Personal Engagement

All informants stated that they or their organisation had a role to play in containing antibiotic resistance. However, many highlighted that Strama (the Swedish strategic programme against antibiotic resistance) was more directly involved in work against antibiotic resistance than their own organisation. A few talked about their personal engagement. One was very frustrated about the low activity to contain antibiotic resistance in Sweden and at the global level.

#### 3.2.2. Legislation, Governance, and Resources

Legislation to protect development of antibiotic resistance in the environment was not an option according to the informants. Legislation was not possible because we do not know which measures to take, said the Environmental Protection Agency informant. The researcher informant did not believe that new regulations were necessary, because factors in society that are important to contain antibiotic resistance, such as functioning healthcare and functioning infrastructure, already existed in Sweden, although they can be improved.

There were different views among the informants on who governs or should govern work to contain antibiotic resistance. One said that healthcare and some social institutions should control the work, whereas another could not see who should be responsible. One informant concluded that there was no central governance in how to conduct the work in wastewater treatment plants. Many had, however, a clear belief that the issue must be on the national agenda. The national government has the resources and must decide what to do, said one informant. 

Several of the informants said it was difficult to determine whether enough resources were available for work to contain antibiotic resistance. One informant observed that pharmaceutical companies needed extra resources for development of new antibiotics, and another informant lifted the necessity of long-term funding to preserve existing antibiotics which were no longer profitable.

Furthermore, financing was a factor that could affect the work of many informants. The researcher was dependent on funding for the research group. Informants from wastewater treatment plants applied for funding for monitoring and developing new treatment techniques. According to the informants, the funders were often national authorities and institutions, which in turn were funded by the government.

#### 3.2.3. Cooperation and One Health

All informants thought cooperation was important, and it was mentioned as a facilitating factor in work to combat antibiotic resistance. Reasons for wanting to cooperate were many, e.g., limited resources, the broadness of the issue and involvement of several sectors, or the fact that when working together, organizations could become stronger and were in a better position to make demands. Some informants emphasized the importance of international cooperation and said it was necessary because problems with antibiotic resistance were greater outside Sweden.

Only a few informants knew about the One Health concept. The government authority informant had a general understanding of the concept, and the researcher informant was familiar with the concept. However, the concept One Health was not known among the rest of the informants.

### 3.3. Informants’ Perceptions of Factors Hindering Work

#### 3.3.1. Difficulties in Setting Environmental Requirements

Setting environmental requirements on pharmaceutical production when purchasing drugs for hospitals, as well as for pharmacies, could be difficult according to informants working in these areas.

“*As I have understood it, so this with procurements is quite tricky when it comes to drugs because it is difficult,* *there is not always good transparency so that you know how this is manufactured […] As I have understood it the pharmaceutical companies are quite like, it’s a bit difficult to get insight into what is done*.”

(M4)

Pharmacy chains cannot influence the production of prescription drugs, informants from this area noted, because Swedish pharmacies must dispense the “product of the period” (i.e., the cheapest available generic of the prescribed drug, decided by the National Dental and Pharmaceutical Benefits Agency, TLV). One pharmacy informant said that the pharmacy chain had tried to influence a national authority to set environmental requirements on prescription medications the pharmacy was required to deliver, but this had failed.

“*They [TLV] refuse and say that we do what the state has ordered from us, that, they are a state authority, and they try to follow the guidelines and requirements that they have from the state*.”

(M7)

Nearly all informants remembered and mentioned the detection of high levels of antibiotics in a river close to a pharmaceutical manufacturing plant in India. The pharmaceutical company informant, reflecting on this from a supplier’s perspective, said that a single company cannot make demands on the production of the drugs they buy, and all companies must have the same requirements for this to work. There were two possible options, the informant continued: an alliance of all companies with common agreements, or demands of transparency set by a national authority. According to this informant, the company monitors what is financially feasible to implement and then balances the costs of production with the price they can negotiate with the benefits agency in Sweden. 

Maintaining a wide assortment of antibiotics was another challenge. The pharmaceutical company informant explained how the company tried hard to keep the assortment, but this could be difficult due to the fact that the market was shrinking.

“*For this, is the challenge, one of the challenges with existing antibiotics that you have to keep them, they have to exist, there is like no future with them because you want to, you will have reduced use, no money for companies that want to make money*.”

(M10)

#### 3.3.2. Lack of Knowledge

A general problem mentioned by the informants was that knowledge was lacking and it was therefore hard to know which measures to take. For instance, the Environmental Protection Agency had discovered antibiotics in water after treatment in treatment plants, and the researcher informant had been involved in detecting resistant bacteria in water and soil. However, as the informant from the Environmental Protection Agency expressed, we do not know what this means.

“*…[antibiotics] is one of several chemicals, then you see it as a chemical substance. And if it comes out in water and sludge, then you have a cocktail of everything possible, it’s not certain that it is only the antibiotics that play a role, but rather that they interact with everything else that is there […] So it is very difficult to say which role antibiotics play there*.”

(M2)

#### 3.3.3. Lack of Action

Several informants called for actions from the politicians. Political actions were, for instance, requested to influence the pharmaceutical production sites to reduce pollution of antibiotics. Another suggestion was action from the government to initiate drug treatment at wastewater treatment plants. 

#### 3.3.4. Conflicting Priorities

Two informants mentioned how different disciplines sometimes have conflicting priorities. One example was that the hospital hygiene department recommended the hospital to use disposable equipment to reduce infection, whereas the environmental department recommended the hospital to use less disposable equipment for environmental reasons. Another conflict reported by the researcher informant was between physicians and pre-clinical researchers. Physicians want to treat their patients with antibiotics, whereas researchers emphasize the importance of avoiding antibiotics as much as possible. This conflict was also mentioned by the informants from environmental departments. 

### 3.4. Development after the Interviews Were Conducted in 2018

There were four informants who answered the questions. They represented a pharmacy chain, a wastewater treatment plant, the pharmaceutical company, and the Swedish Environmental Agency. Three informants were the same informants that had taken part in the interviews conducted in 2018. The Environmental Agency had a new representative in the cooperation platform against antibiotic resistance, and this person answered the questions. 

The informant from the wastewater treatment plant noted that ozone technology had been developed further and the antibiotic residues which were analysed were effectively removed. However, according to this informant, coordination and cooperation in work to contain antibiotic resistance in the environment had not changed since 2018.

In the field of pharmaceutical products, new activities were mentioned. The work at the pharmacy had not changed, wrote the pharmacy chain informant, and the pharmaceutical company informant reported that the company had continued its work to keeping the broad assortment of narrow-spectrum antibiotic products in Sweden. Other engagements which were mentioned in 2018 had grown. The pharmacy chain had continued its engagement for transparency in pharmaceutical production and was now working together with all Swedish pharmacy chains in achieving transparency in the production of non-prescription drugs. The pharmaceutical company informant reported that the cooperative platform, which had recently been initiated in 2018, now gathered more partners, and the informant was now in the management team. The two informants mentioned new measures taken at policy level. These included governmental assignments for the Swedish Medical Products Agency (MPA) and for the National Dental and Pharmaceutical Benefits Agency (TLV), e.g., to organize an environmental premium for procurement of antibiotics, to strengthen access to older antibiotics, and to open a new knowledge centre for pharmaceutics in the environment, with the aim to spreading knowledge and stimulating measures and development in the area. 

The informant from the Environmental Protection Agency said that there was no major difference in how the agency was monitoring the environment compared to the year 2018. However, the antibiotic resistance issue had started to reach networks and platforms where the Agency was involved, and during 2018 to 2023, the agency will be distributing grants for drug treatment at wastewater treatment plants. The Swedish platform for agencies has a role to coordinate the work to combat antibiotic resistance, but the antibiotic resistance issue was still no big question at any environment agency, reported the informant.

## 4. Discussion

This study explored how a selected number of stakeholders from six different health and public service sectors and areas looked upon their role in combating antibiotic resistance. We found that all of them thought they had a role to play, and that their actions can be described in five subcategories: monitoring environment; developing knowledge; spreading knowledge; reducing antibiotics and bacteria reaching the environment; and activities for restrictive antibiotic use. Many informants were active in more than one of these subcategories. Another finding was that most of them felt a lack of governance in this work. In spite of this, their actions were in accordance with measures suggested in the Swedish national action plan. The One Health approach was only known by the policymaker informant and the medical microbiology researcher, but not among informants at the practical level.

Bloomer and McKee [20] suggest four actions to reduce antibiotic resistance in the environment. The first is prevention to reduce the need for and use of antimicrobials. The other three are different actions to prevent or reduce antimicrobials contaminating the environment: improved/alternative wastewater treatment processes; reduced API (active pharmaceutical ingredient) emissions by manufacturers; and management of manure. The stakeholders in the present study were active in all these suggested activities except the management of manure.

This is the third interview study from our research group exploring how stakeholders from different sectors and areas, and from various levels, perceive their role and how they can contribute to containing antibiotic resistance. In total, 34 interviews were performed during a period of six months in the year 2018. At the time of analysis, interviews were divided into three parts: the human sector; the animal sector, and the environment sector. The rational for this was our perception that the work to contain antibiotic resistance were mainly going on in the sectors separately. Our first two studies focused on human medicine and animal production, respectively [18,19]. In both these areas, work to contain antibiotic resistance had started early [21], and extensive efforts within the sectors had resulted in well informed practitioners and low levels of antibiotic resistance in an international perspective [22]. Further similar findings in both studies were the common belief among stakeholders that antibiotics should be used restrictively, and that there were obvious leaders in each sector which were known by stakeholders at the practical level [18,19]. The leaders they identified represented the national level, and their methods were to provide information and the best available knowledge on how to act to combat antibiotic resistance, i.e., using non-authoritative networks to reach stakeholders to gain change.

Our findings in the present study were different. All informants thought they had a role to play to combat antibiotic resistance and talked about activities they were involved in. However, they did not see any leadership of actions. Some of the stakeholders in our study expressed a personal engagement and they followed new findings from research in their area. This could probably explain why their actions were in line with the recommended actions and appeared to be coordinated according to the national action plan, even though they themselves did not seem to be aware of this plan. These stakeholders seemed to be ahead of the national plan in their requests for actions from authorities. A few years later, some of these requested issues were included in the updated national action plan. Other stakeholders in our study were waiting for information from the policy level. One example was the Swedish Environmental Protection Agency which was awaiting directions from the government to act. 

The Swedish Environmental Protection Agency could possibly take a role to lead the activities to combat antibiotic resistance in the environment. However, the agency had at the time of the interview just recently been involved in the national network of agencies against antibiotic resistance, and had not yet found its role. As the informant explained, they needed more knowledge about what it means when antibiotic residues are found in the environment, and about which measures to take before they can act. In the autumn 2021, the agency reported that the issue of antibiotic resistance in the environment still was no big question at environmental agencies, but that this question had started to reach the networks and platforms the agency took part in. Our findings are in accordance to the global situation in general, and less attention has been given to antibiotic contamination of the environment [20,23]. 

A common finding in the three interview studies was that the One Health approach, with few exceptions, was only known at the policymaker level. Swedish agencies have worked together since an intersectoral coordinating mechanism was initiated in 2012 [21]. This means that One Health at the policy level can be perceived as implemented in Sweden. In contrast, professionals at the practical level of the three sectors we have studied were not at all familiar with the concept. Although the Swedish national strategies adopted cross-sectorial work at an early stage in the year 2000 [21], this strategy has not reached the practical level. However, it is possible that practitioners do not have to know about One Health to be successful in their own field to combat antibiotic resistance, as long as they know what measures to take. It is most likely therefore that cooperation between sectors is necessary at the policy level. 

Cooperation between stakeholders at different levels was seen in our study in the animal production sector [19]. To involve different stakeholders at practical levels in the environmental sector, with all the diverse areas that are must be involved, coordination is necessary. Gulati et al. [24] define coordination as the deliberate and orderly alignment or adjustment of partners’ actions to achieve jointly determined goals. Coordination typically involves the specification and operation of information-sharing, decision-making, and feedback mechanisms in the relationship to unify and bring order to partners’ efforts, and to combine partners’ resources in productive ways [24].

The use of antibiotics is the main driving factor for antibiotic resistance development [11,12]. Thus, using antibiotics restrictively can slow the development of antibiotic resistance. This strategy is useful in all sectors, even the environmental sector. Many of our informants in the present study worked actively to restrict antibiotic use, for example, through education of medical students and spreading knowledge to the public. Being restrictive includes using narrow-spectrum antibiotics when possible, a perception shared by several informants, and in general accepted in Sweden, where narrow-spectrum penicillin is effective, and thus is often used [25]. Access to narrow-spectrum penicillin is therefore important in Sweden. However, there are large global differences in antibiotic prescribing by doctors, drug dispensing by pharmacists, as well as expectations from the public regarding antibiotics. Promoting restrictive antibiotic use, and making sure the quantity of prescribed antibiotics does not exceed treatment duration, may be hard to accept in countries where patients self-medicate, or are able to purchase antibiotics without prescription [26].

Informants representing wastewater plants, environmental departments at hospitals, and pharmacies appeared to have a role in reducing pollution of antibiotics and other chemicals in the environment. Even here, collection of pharmaceutical waste can differ globally. A study in Ghana revealed that four out of five hospitals were without separate collection and disposal programs for waste management, and that large parts of the population had unused, leftover or expired medicines at home [27,28]. Rules and regulations for waste management exist in many countries, but little appears to be known about how these rules are followed by healthcare facilities [27]. Wastewater treatment systems also differ globally. Many treatment systems in developing countries are neither successful nor sustainable, mainly because they are copies of Western treatment systems, where no consideration has been taken as to the appropriateness of the technology for the culture, land and climate of the country in question [29]. 

The Environmental Agency monitored toxic compounds in water and soil. However, the identification of antibiotics in treated water by the Environment Agency did not always lead to any action, the argument being that the board needed more knowledge about effective measures before actions could be taken. It is interesting to note that, in this manner, the Environmental Agency appeared to have the least engagement in work to contain antibiotic resistance in the environment. 

Lack of knowledge, and not knowing what measures to take, was considered problematic by other informants as well. Other problems included the inability to set environmental requirements when the pharmacies and hospital purchased medications, and the lack of central governance in how to conduct the work. These findings suggest the need for some sort of governance in work to contain antibiotic resistance in the environment. The Swedish strategic programme against antibiotic resistance (Strama) has played a central role in providing surveillance of antibiotic use and antibiotic resistance in Sweden since 1995. However, as long as there is a lack of knowledge about which methods have the best effect, it is difficult for an organization to lead the work to limit antibiotic resistance in the environment.

Lack of resources and financing could affect the antibiotic resistance work of informants representing pharmaceutical companies and medical research. The pharmaceutical industry is slowly being incorporated into public health efforts regarding which products and how much of them are used [30]. As noted in our findings, there was preservation of existing antibiotics which were no longer profitable and which can affect the spread of antibiotic resistance, and pharmaceutical companies needed extra resources for development of new antibiotics. The medical research informant depended on funding for the research group, a problem that is not unique for just researchers in Sweden. A recent observational analysis of antibacterial research funding in JPIAMR countries (Canada, Czech Republic, Finland, France, Germany, Israel, Italy, Latvia, The Netherlands, Norway, Poland, Romania, South Africa, Spain and Sweden) showed that only 3% of research projects on antibiotic resistance proposed to tackle issues related to the environment [31].

The first Swedish national action plan with a One Health approach was launched in 2016 [9]. However, the need for multisectoral collaboration in work to contain antibiotic resistance was not new at that time. Cross-sectorial work was mentioned in 2000 in a proposal for a Swedish national action plan [32]. A proposition presented in 2005 included work in human medicine and veterinary medicine, agriculture and food production, and proposed the mapping of environmental effects of antibiotic use in order to learn more about consequences of antibiotic pollution [33]. Still, in the national plan from 2016, focus was set on gaining more knowledge [9]. The plan concluded that knowledge was incomplete, but data indicated that antibiotics and other antibacterial agents in the environment could give rise to antibiotic resistance. Technology for the cleaning of pharmaceutical residues and other substances in water treatment plants should be tested and evaluated. Furthermore, the plan included development of support for county councils’ procurement processes in order to move towards minimizing releases of antibiotics into the environment during the production of pharmaceuticals. 

Strategies were further developed in the updated Swedish national plan from 2020, and issues that the informants in our study asked for have now been included [10]. Now the plan concludes that knowledge of the role of the environment in the development and spread of antibiotic resistance is increasing. Examples of proposed actions include advanced treatment of wastewater, and that Sweden pushes for the development of regulations to steer towards minimized emissions of antibiotics to the environment in pharmaceutical production. According to the updated information given by informants in autumn 2021, some of these proposals have been developed into action by new governmental assignments to the Swedish Medical Products Agency (MPA) and to the National Dental and Pharmaceutical Benefits Agency (TLV).

### Strengths and Limitations

The main contribution of this qualitative study is its insight into the unique perspectives and perceptions of work to contain antibiotic resistance in the environment among a select number of informants working in areas that were supposed to have impact on environmental issues in Sweden. All informants perceived they had a role to play in containing antibiotic resistance in the environment. Our intention was not to present a representative survey of all stakeholders’ perceptions of work to contain antibiotic resistance in the environment. Our findings are thus based on the perceptions of a small sample size of informants and cannot be generalized. It was beyond the scope of this study to include perceptions of other stakeholders in other regulatory bodies or sectors also involved in work to contain antibiotic resistance in the environment. For example, there were no stakeholders from the animal sector included in this study. If other sectors than the chosen had been included, we would probably have identified other activities to reduce antibiotic resistance and resistant bacteria in the environment. A strength of the study is the qualitative design with interviews that allowed the informant to speak freely around the subject. This design gave us a rich material from each of the informants and good insights into their work from their perspective. The analysis was careful and structured to ensure trustworthiness, and was performed by two experienced qualitative researchers. The findings were further strengthened by the follow-up questions sent to the informants and the answers that were given from informants from four different areas.

## 5. Conclusions

A One Health approach to contain antibiotic resistance means to simultaneously work to reduce the development and spread of antibiotic resistance in humans, animals, and in the environment. So far, there appears to be little coordination in the work to contain antibiotic resistance in the environment in Sweden. The stakeholders at the practical level were involved in activities in their own area which they perceived could have an impact on antibiotic resistance, but did not feel that they were included in a common program. Their actions seemed to be coordinated, but this was, according to the stakeholders, based on findings from research in their area rather than on strategies developed by national authorities. The One Health approach has been implemented and was known at the policy level in Sweden, but was not established at the practical level.

## Figures and Tables

**Table 1:** The informants and the rational for choosing the selected area of work.

Health and Public Service Area	Informants	Rational for Choice of Selected Area
Government authority	One analyst working at the Swedish Environmental Protection Agency	According to the agreement taken in the WHO, national governments should develop national action plans to combat antibiotic resistance. In Sweden, a national action plan was ready in 2016, and updated in 2020 [9,10]. Even so, written strategies must be put into practice, and authorities have an important role to stimulate action on behalf of the government.
Microbiology research	One researcher in medical microbiology and genetics	There is a need for new knowledge to understand the role of the environment in antibiotic resistance development and spread. Research in multiple fields is necessary, and one of the fields is medical microbiology and genetics. The focus of this researcher was basic research on how resistant genetic material is transmitted.
Pharmaceutical companies	One pharmaceutical company representative working in the company’s medical department	The pharmaceutical industry plays an active role in research, discovery, and development of new drugs and medicines. It also has impact on production methods, and on the availability of drugs and medications on the market.
Pharmacies	Two pharmacy representatives responsible for quality management in their respective pharmacy chain	Consumers of antibiotics purchase their medications at a pharmacy. In addition to dispensing medications prescribed by physicians, pharmacists and pharmacy technicians can influence how consumers manage medications they purchase at the pharmacy. Another role for pharmacies is to collect consumer medical leftovers.
Hospitals	One environmental scientist working in a regional environmental department, responsible for environmental issues in hospitalsOne hospital environment department head.	Hospitals are major users of antibiotics and preventing pollution from hospitals seems to be essential. Patients in hospitals suffer from more complicated infections and are often treated with multiple and or broad-spectrum antibiotics. Many antibiotics used by hospital patients leave the body unmetabolized and end up in the wastewater.
Wastewater treatment	Two water treatment plant representatives responsible for municipal water quality control in two major Swedish cities	Wastewater plants are receivers of city wastewater, and their role is to remove undesirable chemicals and microorganisms, or reduce their concentration, so that water becomes clean enough to be released into the environment. They also treat sludge from wastewater, which after treatment is often used in agriculture.

**Table 2:** Interview guide, main questions.

1. What does antibiotic resistance mean to you?
2. How do you look upon your role in working to contain antibiotic resistance?
3. How do you look upon possibilities of limiting/preventing emergence and spread of antibiotic resistance?
4. What do you think are the main causes of antibiotic resistance?
5. How do you think antibiotic resistance spreads?
6. How do you look upon the use of antibiotics in humans, animals, or any other areas?
7. Have you heard of the concept of ’One Health’?
8. Do you have any comments to add?

**Table 3:** Main categories and subcategories describing the content of the interviews.

Categories	Subcategories	Area of Work Involved
Informants’ examples of actions taken to combat antibiotic resistance	Monitoring and risk analysis	Government authorities
	Developing knowledge	Medical microbiological research Wastewater treatment
	Spreading knowledge	Medical microbiological research Pharmacies
	Reduce antibiotics and bacteria reaching the environment	Pharmacies Hospital environment department Wastewater treatment
	Activities for restrictive antibiotic use	Medical microbiological research Pharmaceutical companies Pharmacies Hospital environment department
Informants’ perceptions of factors influencing work	Organisational and personal engagement Legislation, governance, and resources Cooperation and One Health	All areas of work contributed here.
Informants’ perceptions of factors hindering work	Difficulties in setting environmental demands Lack of knowledge Lack of action Conflicting priorities	All areas of work contributed here.

## Data Availability

The datasets presented in this article are not readily available because they consist of in-depth key informant interviews containing sensitive participant information. Due to the small number of persons in this field in Sweden, it may be possible to deduce the identity of the interviewee, which would violate the anonymity agreement with the participants. Requests to access the datasets should be directed to Jaran Eriksen, jaran.eriksen@ki.se.

## Supplementary Materials

The following are available online at https://www.mdpi.com/article/10.3390/antibiotics11050646/s1, File S1: Swedish Interview Guide for policy level and professional level.
