# Supplementary material for: Swedish Efforts to Contain Antibiotic Resistance in the Environment—A Qualitative Study among Selected Stakeholders
Source: Antibiotics (Basel). 2022 May 12;11(5):646. doi: 10.3390/antibiotics11050646 (PMC9137522; doi:10.3390/antibiotics11050646)
Supplement: Supplementary file 1 [file antibiotics-11-00646-s001.zip › antibiotics-1692291-supplementary.pdf]

Coordination, Accountability, Resourcing, Regulating and Ownership of ABR: A One Health System and Policy Approach (ABR- CARRO)
Swedish Interview Guide for policy level and professional level

Policy makers/government level

1. What do you understand by antibiotic resistance?
   Probe:
   a. Is ABR a problem? If so, for whom, where and why?
   b. What do you think are the consequences of antibiotic resistance?
2. What is your role in antibiotic resistance containment?
3. What is your view on the ABR containment?
   a. governance (coordination, accountability)
   b. resourcing
   c. regulation
   d. ownership
   e. implementation
   f. monitoring and evaluation
   g. human, animal and environment
4. What do you understand by one health? How do you define the concept?
   Probe:
   a. Human, animal and environment
5. What do you think needs to be done to prevent and contain ABR?
   Probe:
   a. Vaccination, infection prevention, bio security, waste disposal, water treatment….
6. What do you think are the most important barriers & enablers to ABR containment?

Professionals

1. What do you understand by antibiotic resistance?
   Probing:
   a. Is ABR a problem? If so, where and why? In your own area or otherwhere?
   b. What do you think are the consequences of antibiotic resistance?
2. How do you see antibiotic use in humans and animals, and other areas?
   Probe:
   a. Community vs. hospital use in humans, use in viral infections,
   b. Growth promoters, prophylaxis and metaphylaxis in (food) animals.
   c. Use in agriculture.
   d. Disposal? Residues in environment?
3. What do you think causes antibiotic resistance?
   Probe:
   a. Indiscriminate use (overuse, underuse, misuse)
   b. Hygiene
   c. Use as growth promoters, prophylaxis and metaphylaxis in (food) animals.
   d. Environment
   e. Biocides, metal.
4. What do you think causes antibiotic resistance to spread?
   Probe:
   a. Infection prevention and control in human health.

    b. Biosecurity in (food) animal health.
    c. Use of manure-based fertilizers, water contamination.
    d. Spread along the food chain.
5. What is your view on the ABR containment?
    a. governance (coordination, accountability)
    b. resourcing
    c. regulation
    d. ownership
    e. implementation
    f. monitoring and evaluation
    g. human, animal and environment
6. What do you think is your role in preventing/containing antibiotic resistance?
